# Observations on Extra-Uterine Cases, and on Ruptures of the Uterus

**Published:** 1787

**Authors:** Maxwell Garthshore

**Affiliations:** Fellow of the Royal College of Physicians at Edinburgh, and Physician to the British Lying-in Hospital in London


II. *Obſervations on Extra-uterine Caſes, and on Ruptures of the Uterus. By* Maxwell Garthſhore, *M. D. F. R. S. and S. A. Fellow of the Royal College of Phyſicians at Edinburgh, and Phyſician to the Britiſh Lying-in Hoſpital in London.*

THERE are few things more curious, and fewer ſtill that are more uſeful, for an attentive phyſician to obſerve, than the very wonderful reſources of which nature, undiſturbed, is ſometimes able to avail herſelf, when labouring under diſeaſes ſeemingly deſperate. Amidſt the numerous variety of examples of this kind, which the curioſity and induſtry dustry of writers of observation have recorded, there are none more striking than those means she has frequently made use of to free herself of the burden of an extra-uterine child. The great number of modern as well as ancient well-authenticated cases, in which the child has made its way, piecemeal, either through the ulcerated integuments of the abdomen, or the coats of the intestines, with safety to the mother, may shew us that the existence of a child in the open cavity of the belly, and its subsequent resolution, or its being turned into an indurated mass, is not always so fatal as might be apprehended; and what is still more extraordinary, we are not without instances where this process has taken place when the conception was not originally extra uterine, but where in the latter months of pregnancy the uterus has been ruptured, and the child has escaped into the cavity of the abdomen. Of this we read several examples in the curious Dissertation of Thomas Bartholine, *de insolitis partus humani viis*, published at Copenhagen in 1664; in all of which the uterus was evidently ruptured, either in the latter end of pregnancy, or in labour, and the child had evidently passed from the womb into the abdominal cavity, and afterwards made its way through the integuments of the abdomen, or by the inteftines *.

To this clafs I think may be alfo referred the cafe of a woman communicated by Dr. Percival in the Medical Commentaries, (Vol. II. page 77,) where, from the circumftances of flooding and pain brought on by a fudden fright in the fixth month of pregnancy, there is great reafon to fufpect that the child then efcaped into the general cavity, from which it was afterwards expelled, piecemeal, by the rectum, at the end of twenty-two years. This woman's body not having been examined after death, leaves the fceptical room to doubt whether any actual rupture took place; but we have a more decifive proof of the poffibility of fuch an event in the cafe of the woman of Touloufe, mentioned by Aftruc †, who, during the pains of a very laborious birth, had her uterus burft, and her child paffed into the cavity of

* In two of the four cafes of this kind, which he mentions, the fœtus made its way through the integuments of the abdomen, and in the two others by the inteftines; three of the women recovered entirely, and the fourth furvived fome time, and paffed many of the bones of her fœtus by ftool, but did not live long enough to get rid of the whole.

† L'Art d'Accoucher, Chap. iv. page 288.

the abdomen, where it remained for twenty-five years, as was demonſtrated when her body was examined after death.

In the Hiſtory of the Royal Medical Society at Paris* we have a ſimilar caſe communicated by M. Deſbois, of a woman at Rochefort, mother of three living children, who, in her fourth labour, after ſuſtaining pains of the moſt excruciating kind for thirty hours, and when, to all appearance, the birth was nearly effected, had at once the uterus burſt through, and the child paſſed into the cavity of the abdomen. Her pains from that moment left her entirely, and ſhe felt only a dead weight in the hypogaſtric region. Fifteen days after, being examined by M. Rochard, Surgeon, he could find no part of the child, the head of which had been ſo plainly felt by the midwife during the whole of the labour. After two months the integuments of the abdomen began to inflame, and there very ſoon broke out four different ulcers, which emitted very fœtid, purulent matter. In the third month after the inflammation had begun, when the woman was ſinking under colliquative ſweats and hectic fe-

* Vol I. for 1776, page 388. 4to. Paris, 1779.

ver, ſhe was brought to the Hotel Dieu at Paris, and from the largeſt of theſe abdominal ulcers, which was dilated, the bones of a full grown child were extracted. In four months this woman had recovered her wonted looks and ſtrength, and had no complaint but a fiſtulous ulcer at the navel, which emitted not only white, purulent matter, but ſometimes even the fæces, a clear proof that ſome part of the inteſtinal canal had been ulcerated, and adhered to the peritonæum.

A caſe very ſimilar to this is inſerted in the *Journal Encyclopedique* for June, 1777; and another was communicated formerly to the Royal Academy of Sciences* by M. Littre, with this difference, that the bones of the child, in that inſtance, made their way through the rectum.

In the volume† I have juſt now quoted, of the Hiſtory of the Royal Medical Society, a caſe ſo very extraordinary is communicated by M. Bouillon, Phyſician at Mortain, that nothing but the reſpectability of the publication in which it is inſerted would induce me to copy

* Mem. de l'Acad. Royale des Sciences, année 1702, p. 234.

† Page 310.

it.

it. We are there told that a well-formed woman, mother of many children, was attended in a preternatural labour by a very unſkilful accoucheur, who, after many fruitleſs attempts, at laſt brought away the child dead, but with only one arm, the other having been left in the body of the mother. She continued, after this, to have pains; fever ſoon came on, and an inflammatory tumor was formed in the hypogaſtric region, which ſuppurated, and diſcharged a large quantity of purulent matter; and ſoon after the humerus, and in ſucceſſion the other bones of the upper extremity, which, during the labour, had been ſeparated from the trunk of the fœtus, preſented themſelves at the abſceſs, and were extracted, and the woman by mild dreſſings was completely cured.

In the *Journal de Medecine*, (Vol. VI *.) we have a caſe communicated by M. Guillerme, of a ſtout woman, who, in her thirty-firſt year, by a fall from a waggon, had the uterus burſt in the fifth month of pregnancy, which was ſucceeded by flooding, fever, and violent pains. The os uteri was found open, but no abortion took place. The child was from that moment without motion, and her belly diminiſhed in

* Page 292.

ſize.

ſize. In two months ſhe recovered ſeemingly her uſual health, and remained well till the ſeventh month after the accident, when there aroſe an inflammation of the abdomen, attended with acute fever, and ſucceeded by a violent diarrhœa, in which ſhe paſſed great quantities of fæces, ſo very fœtid, that ſhe was obliged to be removed to a room by herſelf. In the eighth month from the accident, and when this diarrhœa had continued ſome weeks, ſhe began to paſs the bones of a fœtus, ſeemingly of five months, and continued ſo to do for the three ſucceeding months, and before one year had elapſed, from the time of the accident, ſhe was reſtored to perfect health. But a ſtill more certainly-authenticated caſe of the ſame kind happened lately in this capital:—A poor woman, after violent exerciſe, ſuffered a rupture of the uterus in the ſeventh month of pregnancy, and ſurvived it ſo long as to give nature an opportunity of completely encloſing the excluded fœtus in a ſtrong ſac, from the bottom of which all the ſoft parts had gradually paſſed into the lacerated uterus and through the vagina; and when the bones had begun to make their way through the ſubſtance of this ſac and the abdominal integuments, the ſalutary tary proceſs of nature was interrupted, a new and fatal inflammation brought on, and the woman deſtroyed, by an imprudent expoſure to violent motion above four months after the rupture happened. Need I add the authority of Plenck, who, treating of rupture of the uterus, mentions the two reſources of nature, already deſcribed, in the following words*: — "Moriuntur infelices hæ matres utplurimum "intra aliquot dies ex uteri, et abdominis gan- "græna..... Interim tamen habentur caſus, "quibus fœtus extra uterum lapſus per abſceſ- "ſum, vel gangrænam topicam abdominis "exierit, et mater fuerit ſervata..... Poteſt, "et fœtus in lithopædion mutari, et gravidi- "tatem perennem inducere."

In farther proof of the wonderful power of the vis medicatrix naturæ, nothing can be ſtronger than the circumſtances of the very curious caſe communicated to Dr. Simmons by Dr. Underwood, the conſideration of which led me to this inquiry, as I am inclined to believe that the uterus in that caſe was ruptured when the patient fainted from the violence of the ſhock ſhe received at the end of the fifth month,

* Elementa Artis Obſtetr. page 129. 8vo. Viennæ, 1781.

and and that to this rupture were owing all thofe violent fymptoms which afflicted her for the five fucceeding years. But what adds very much to the fingularity of the cafe is, that there is much reafon to fufpect, from the enlargement of the abdomen, fecretion of milk in the breafts, and the other fymptoms defcribed by Dr. Underwood, that this woman became, at the end of feven years, a fecond time pregnant, and whilft the former fœtus remained in the abdomen; and this fecond conception, if it did take place, was moft probably extra uterine. With this fhe was haraffed for fix years more; and it was not till the twenty-firft year after the fuppofed rupture that fhe began to get rid of thofe fœtal bones by the inteftines, which continued to be difcharged with infinite pain and diftrefs for above eighteen fucceeding years; and from their great number, and the length of time neceffary to their expulfion, we have ftill ftronger reafon to fufpect there was a fecond conception. But we want the decifive proof, which could only have been obtained by the examination of the body after death. I am, however, juftified in the probability of my conjecture by examples, of which there can be no doubt.

In

In the second volume of Medical Observations and Inquiries, Mr. Bard, of New York, communicates a case, to the termination of which the late Dr. Huck Saunders was witness, where a woman, who had been fourteen months pregnant of an extra-uterine child, conceived a second time, and was delivered at the full term of a healthy boy; soon after which the abdominal tumor occasioned by the former child began to suppurate, and being afterwards opened, a fœtus of the common size was extracted from it. In the fifth volume of the Medical Essays of Edinburgh, Dr. King, of Armagh, communicates a case where a woman, who had been six years pregnant with an extra-uterine fœtus, became with child a second time in the same way. The fœtus of the second conception was extracted almost entire through an ulcer formed in the integuments of the abdomen; and the bones of the first were gradually passed, partly by the rectum, and partly, as is said, by the bladder, by which I suppose is meant the vagina.

But what approaches still nearer to what I suppose to have been the circumstances of Dr. Underwood's patient is the case related by Primerose, rofe *, of a woman at Bourdeaux, who, after having two years carried a fœtus, fuppofed to have burft from the uterus, became pregnant a fecond time of an extra-uterine one. The firft was extracted through an abfcefs formed in the abdominal integuments, and the fecond by an operation performed on the other fide of the abdomen. Thomas Bartholine, who quotes this cafe in his Differtation before mentioned, and who confiders all the concomitant circumftances at great length, entertains no doubt that the former of thefe children had made its way through the uterus into the open cavity. But fhould his reafons not appear fufficiently fatisfactory, we have ftill a more unequivocal cafe in the *Journal de Medecine*, (Vol. V †.) communicated by M. Bochard, of a woman in Dauphiny, who, in the feventh month of pregnancy, by a fall from a tree, had the uterus burft, and the child paffed into the open cavity, from which time fhe felt no more motion, and was difcovered, on examination fome months after, to have a heavy, moveable tumor in the abdomen, and to have fuffered much pain and inquietude. At the end

* De Mulierum Morbis. 4to. Roterod. 1655. p. 326.

† Page 422.

of the fifth month from the accident ſhe had diſcharges of blood, from the uterus, for ſeveral days together, mixed with portions of hair, of which ſome were evidently of the head, and in a week after theſe diſcharges her abdomen was conſiderably diminiſhed in ſize. In the ſeventh month from the fall ſhe became again pregnant, and in the ſeventeenth was delivered of a living child. Three months after this (that is about twenty months after the fall) a painful tumor was formed in the integuments of the abdomen, and very ſoon after an open ulcer, from which much purulent matter was diſcharged, and afterwards ſmall bones. This induced M. Glodat, the Surgeon, who was concerned in the caſe, to dilate the opening, from which he extracted the ſkeleton of a child, and the placenta in a ſeemingly petrified ſtate. This woman, as well as the three former I have mentioned, are all ſaid to have been reſtored to perfect health.

If I have in any degree been able to ſatisfy the curious that nature is ſometimes able to free herſelf of a fœtus that has made its way through the uterus, I certainly ſhall have leſs difficulty to prove that ſhe may more eaſily free herſelf from the burden of a child formed in the general cavity, and there would be no difficulty in producing inſtances of that kind, but I am certain they are already ſufficiently known to all who inquire into theſe matters. I cannot, however, refrain from mentioning one, as being the moſt extraordinary I have ever heard of. It is quoted by Bianchi * from Ruleau's work on the Cæſarean operation, and relates to a woman who became pregnant, at three different periods, of three ſeparate extra-uterine children, which all died in the early months of pregnancy, all became putrid in the body, and were all extracted through an ulcer formed in the umbilical region. Bianchi conciſely adds, "Hæc igitur mulier ter partuum labores, ter internos abortus, ter fœtuum neces, terque eorum corruptiones ſuſtulit; deinde ſana rectéque fœcunda vitam degit," by which it appears that this woman not only completely recovered, but became fruitful afterwards. The inſtances in which women have ſurvived, for many years, extra-uterine children, by their being converted into what has been called a petrified or cartilaginous maſs, are too well known, and have had too much ſaid about them, to make either the

* De naturali in human. corpore vitioſa morboſaque Generatione. 8vo. Turin, 1741. p. 100.

fact fact doubtful, or quotations necessary; yet I can hardly pass over the instance of this kind given us by Dr. Starkey Myddelton, in the Philosophical Transactions*, of a woman he opened at Guy's Hospital in 1747, in whose abdomen he found the body of a child attached to the right fallopian tube, and changed into a kind of cartilaginous mass. This woman he had formerly attended with Dr. Bamber, and was well assured that sixteen years before, this extra-uterine child had died in consequence of a fright in the sixth month of pregnancy; that, after suffering much distress, the woman, at the end of twenty-six months, had become a second time pregnant, been delivered of a living child, and afterwards of three more at the full term, and had certainly carried this extra-uterine fœtus full sixteen years before her death.

To this instance I could easily add several more of what have been, though improperly, called petrified children, which have remained in the body from five to forty-six years, without much disturbing the health of the mother, who, in some of the instances, bore (as in the case above mentioned) healthy children in the time whilst this mass remained.

* Vol. XLIV. page 617.

But

But the moſt extraordinary inſtance of this kind is that of the woman at Turin, in whoſe abdomen Bianchi*, in 1728, found a fœtus, weighing eight pounds, which had, as he ſuppoſed, fifteen years before, burſt from the right ovarium at the full term of pregnancy, and was found covered very cloſely by the membranes, and incloſed by a thick ſebaceous cruſt, which, on being expoſed to the air, hardened into a kind of gypſum; by which cruſt, and the membranes of the ſecundines, the child was preſerved ſo freſh, and flexible, as to exhibit the appearance of a mature child not long dead.

In a table, which I have now before me, of ſixteen extra-uterine caſes, extracted from the moſt reſpectable authorities, I find that ſeven of them terminated, as in Dr. Underwood's caſe, by the bones of the fœtus having paſſed off by the rectum, and that in the nine others the fœtus was extracted through abſceſſes formed in the integuments of the abdomen, and that moſt of the women recovered entirely, and ſeveral of them bore healthy children afterwards.

I ſhall now beg leave to offer ſome obſerva-

* De nat, in hum. corp. vitioſa morboſaque Generatione, p. 166.

tions tions relating to conceptions in the ovaria and fallopian tubes, and to the ruptures which are their general confequence. I am induced to do this by a cafe defcribed lately in the London Medical Journal * by Mr. Jacob, of Faverfham, which, in fome refpects, is ftill more curious than the one communicated by Dr. Underwood, which I have been juft now treating of. I have no doubt but the conception, in Mr. Jacob's patient, was originally formed in the fallopian tube, and I am led to be of this opinion from a confideration of the inceffant colic, dyfury, coftivenefs, ficknefs, and want of natural reft, with which fhe was harraffed in the early months of pregnancy; and ftill more decifively from that inward fenfe of plunging, attended with fevere pain, which continued without ceffation, and was fo violent as to bring on convulfive fits, in the fixth month; it being perfectly well known to thofe who have attended to the fymptoms of ovarian and tubal conceptions, that they are always attended by many irregular, painful, and diftreffing complaints during the whole term of pregnancy; and that they are peculiarly diftinguifhable from uterine conceptions, by thefe complaints con-

* Page 147.

tinuing tinuing to increaſe in violence in proportion as the pregnancy advances, and this from the moſt obvious cauſes, viz. the violent and preternatural diſtention of the narrow ſpace in which the ovum is unfortunately lodged, and from the irregular compreſſion they often occaſion to the abdominal organs: whereas in uterine conceptions, in the latter months of pregnancy, all theſe anomalous ſymptoms, which are ſo common and diſtreſſing during the early months, are, after the fourth or fifth, greatly alleviated, and moſt commonly totally diſappear. But had this conception not been lodged either in the ovarium or tube, the patient could hardly have eſcaped abortion under ſuch violent ſymptoms, as that accident occurred in her firſt pregnancy from a very ſlight cauſe; and what ſtill farther confirms its being a tubal conception is, her having experienced the common termination of ſuch caſes by the rupture of the tube in the ſeventh month, which appears to me to have happened when "ſhe ſuddenly waked in a "great fright after having dreamed ſhe had "fallen from a precipice," as from that time ſhe not only found an evident alteration in the ſituation, but alſo a total want of motion, of the child, which ever after felt to her like a dead dead weight in the abdomen. I, therefore, think it is more than probable that the child then paſſed into the abdominal cavity, and that this rupture, together with the conſequent alteration of the child's ſituation, and its death, gave occaſion to all that ſubſequent variety of ſymptoms with which this poor woman was haraſſed until the twenty-fifth month after conception, and the eighteenth from the ſuppoſed rupture, when the fœtus was extracted through an abſceſs formed five months before in the integuments of the abdomen. If we may be allowed to judge by the growth of fourteen inches, to which only this child had arrived, we muſt ſuppoſe either that it had been very much retarded in its increaſe by the ſac in which it was contained, or that it muſt have died previous to the ſeventh month, when it burſt its way into the general cavity.

When we attend to the circumſtance of the menſes, (which have been conjectured, with ſome probability, to depend on the ſtate of the ovaria) we muſt be led to ſuppoſe, that when they appeared in the ſeventh month after the rupture, and the thirteenth after conception, the uterus and its appendages were diſengaged from the fœtus; and had not their regular diſ-

charge, after ſeven ſucceſſive periods, been obſtructed by the derangement of the conſtitution from the abdominal inflammation, which then ſupervened, it is probable they might have continued to be regular till ſhe had again become pregnant.

A woman ſurviving this accident is, perhaps, a rarer occurrence than her ſurviving a rupture of the uterus; as it has been clearly aſcertained that conceptions in the ovaria or Fallopian tubes are not only diſtinguiſhed by the exquiſite and increaſing pain which attends the whole progreſs of ſuch geſtations, but by the ſudden fatality which the burſting of the ſac generally occaſions. Of this we have a variety of inſtances very well authenticated. Among ſeveral recorded in the Philoſophical Tranſactions, there is one very remarkable, deſcribed by M. de St. Maurice *, of a woman who died ſuddenly in conſequence of the fœtus burſting from the right ovarium; and another, communicated by Dr. Fern †, in which the fœtus burſt from the tube, and which was equally fatal. M. Chambon de Montaux ‡

* Phil. Tranſ. No. 150, p. 285.

† Ibid. No. 251, p. 121.

‡ Des Maladies de la Groſſeſſe, Tome II. p. 373.

mentions the cafe of a woman who died very quickly in confequence of an effufion of blood occafioned by a conception of two months having fuddenly burft from the right ovarium; and in the firft volume* of the Medical Commentaries of Edinburgh a cafe is mentioned, which Dr. Hunter ufed to relate in his Lectures, of a woman, in whofe body he had found the Fallopian tube burft by the growing conception in fo violent a manner as to occafion a fatal internal hæmorrhage. But we have had a ftill more remarkable cafe, of a fimilar kind, which happened lately at an hofpital in this city, of a woman who was admitted under fufpicion of a dropfical complaint, but was difcovered to be pregnant, and who, after fuffering infinite diftrefs, was at laft deftroyed by the gradual dilatation of the left Fallopian tube, in which a fœtus had been conceived, and had advanced to the end of the fifth month of pregnancy. In this cafe, although the fœtus had not yet burft into the general cavity, yet it had rendered the tubal fac fo thin and tender, that it could not bear handling, but gave way on one fide when the parts were taken out of the

* Page 429.

body, ſo as to diſcover nearly half of the child. This curious preparation is now in the collection of Mr. Watſon, who has been long accuſtomed to anatomical inquiries, and who means to give a more particular account of it. This caſe, as well as that mentioned by Boehmer *, where the mother died when the child had only paſſed one leg through the lacerated ovarium in the fourth month, may ſhew us that the hæmorrhage, which ſeems to have been the immediate cauſe of death in the caſes I have already mentioned, is not always a neceſſary circumſtance, as the inexpreſſible anxiety, pain, fever, and inflammation, that are commonly brought on, are of themſelves ſufficient to deſtroy.

Though this burſting of the fœtal ſac will generally take place before the end of the fourth month, and that more readily if the conception be formed in the ovarium, which does not ſo eaſily yield to the increaſing bulk of the fœtus, yet we ſee that conceptions in the Fallopian tube do often allow of a greater dilatation; nay, we are told of ſome rare caſes where they have advanced, in this ſituation, till the full term of pregnancy. One of theſe

* Obſerv. Anat. Faſcic. I. Fol.

is quoted by Haller, and another by M. le Roux *, of Dijon †; yet M. Baudelocque's account of the matter ‡ ſeems to me to be founded on good obſervation.

Though the caſe of a fœtus making its way from the Fallopian tube, or ovarium, is very commonly fatal, either immediately, or in its conſequences, yet we are not without inſtances where, as in the caſe related by Mr. Jacob, the woman has ſurvived. Pouteau § tells us of a Madame Claris, who, at an advanced age, had a conception formed in the left Fallopian tube. In this caſe all the ſoft parts of the fœtus were paſſed off by ſtool, and the bones,

* Obſ. ſur les Pertes de Sang. p. 24.

† It appears, notwithſtanding theſe exceptions, that the Fallopian tube cannot, in general, ſuſtain a dilatation greater than is ſufficient to contain an infant of three or four months, and that it is at this period that the fœtus uſually periſhes; after which it either becomes dry or putrid. Sometimes alſo the tube gives way, and the child eſcapes into the abdomen, where it undergoes one of theſe changes. In all theſe caſes the fate of the woman is different, according to the change the child undergoes. She may live a long time, and without her health being much affected, when it is dried, or indurated, as it were, but ſhe will certainly feel very dangerous effects when putrefaction takes place.

‡ L'Art des Accouchemens, Tome II. p. 329.

§ Melanges de Chirurgie, p. 383.

after after death, were found lodged in the tube, after having remained there ſeven years and a half. During the whole of this time ſhe ſuffered great diſtreſs; and it is more than probable ſhe might have ſurvived the complete evacuation of the fœtal bones, had it been poſſible to prevail on her to take the leaſt care of her health.

Cyprianus * formerly publiſhed a very remarkable caſe of a lady who had a full grown fœtus, which had been originally contained in the right Fallopian tube, and which occaſioned an abſceſs in the abdominal integuments, through which it was extracted, in the twenty-firſt month after conception; and the patient recovered ſo completely as to be afterwards the mother of three living children.

Bianchi † mentions the caſe of a woman, who, at the age of thirty-two, conceived a child, as he ſuppoſed, under the external membrane of the left Fallopian tube, which appears to have burſt from its ſac in the fifth month, and to have occaſioned a variety of complaints

* Hiſt. Fetus humani ſalva matre ex tuba exciſi. 8vo. Leid. 1700.

† De naturali in hum. corp. vitioſa morboſaque Generatione.

in in the ſtomach, inteſtines, and uterus, till the eighth, when ſhe began to paſs the bones by ſtool, and continued ſo to do for the five ſucceeding months. She ſurvived their total expulſion three months, and, after her death, what was ſuppoſed to be the placenta, was found adhering to the poſterior part of the uterus, to the fimbriæ of the right Fallopian tube, and to the colon, where a paſſage was found through which the bones had paſſed,

As the poſſibility of ſurviving this accident ſeems to be well aſcertained, I hope my conjecture of its having been the caſe in Mr. Jacob's patient may not be deemed very improbable, and therefore the urging more reaſons, or quoting more inſtances, may be thought unneceſſary; but as I am upon the ſubject of the female conſtitution being able, by the powers of nature alone, to ſurvive conceptions of this kind, it may not be improper juſt to add one inſtance of the other method by which we have ſaid nature ſometimes evades, as it were, the fatal conſequences that would otherwiſe follow — I mean that in which the child dies early in the ovarium, or tube, and becomes an indurated maſs. The nature of this ſubſtance, which has been mentioned under different appellations, pellations, has never been clearly ascertained; all those instances of it, which I have examined, consisted only of the bony and cartilaginous parts of the fœtus (the soft parts having been previously melted down and carried off) closely compressed together in a sacculus by the surrounding parts, where they became much drier than common cartilage, and were sometimes firmly united by animal mucus, which was also become nearly hard. I have given one very singular instance of this kind, from Bianchi, in the former part of this paper, and I will only add another, which we find mentioned by Dr. Fern in the Philosophical Transactions*, who tells us, that, upon opening the body of a woman who had supposed herself to be three months gone with child, he found the womb not larger than it is usually found in virgins, but a hard substance in the right Fallopian tube, which, being opened, appeared to be the skeleton of an infant, with the navel string smeared round with a white substance not unlike plaster. M. Baudelocque † mentions a very

* Vol. XXI. p. 121.

† Nous y avons rencontré il y a plusieurs années, une masse osseuse assez informe, entourée de neuf dents bien solides, & beaucoup very remarkable appearance in the ovarium, of which there have been other inftances. Roederer * defcribes alfo a peculiar termination of the ovarian conception, but of which I have not met with any other inftance, and that is, its occafioning a dropfy of the ovarium, and that, after tapping, the remains of a fœtus were extracted through the dilated orifice.

It may perhaps be expected that, before I conclude this fubject, I fhould fuggeft a few things refpecting the ufual fymptoms by which we may diftinguifh thefe extra-uterine conceptions; though, in general, it muft be acknowledged, that in the early months they are, and muft be, attended with great obfcurity, as none of the fymptoms, faid to be diftinguifhing, can be depended on till we are able to determine the exact ftate of the uterus by examination.

We are told that in the purely ventral conceptions the menftrua continue regular, that the ftomach is not affected with ficknefs and vomiting, and that there is no fecretion in the breafts; but all thefe circumftances do fomebeaucoup de cheveux entre mêlés dans une grande quantité de matiere, comme butireufe. — L'Art des Accouchemens, Tome II. p. 322.

* Elem. Art. Obftet. Cap. xxv. § 758.

times take place in uterine conceptions. If, however, the child be in the open cavity, it will certainly give lefs pain to the mother, will feel freer in its motions, and be more readily diftinguifhed under the integuments; but as it increafes in fize, in whatever region of the abdomen it happens to be fixed, it muft always occafion painful effects to thofe vifcera that are expofed to its compreffion; fo that either the action of the ftomach, liver, inteftines, kidnies, or bladder, may occafionally be much deranged by an increafing tumor of this kind.

Conceptions in the ovaria or Fallopian tubes will always be confined to the lower region of the abdomen, and the fwelling will always begin on one fide, and muft conftantly be attended with fevere and increafing pain, which is faid to be more acute when the conception is in the ovarium than when it is in the tube. In thefe cafes the menfes are obftructed, and the milk fecreted, as in the cafe of uterine conception. In the ventral conceptions, however, it may not be difficult to diftinguifh, when we examine *per vaginam*, that the uterus is empty; for it is faid to be enlarged even in thefe cafes: yet it would be more difficult to determine in early pregnancy, when the conception is in the ovaria or tubes, whether it is uterine or not, as there muſt be ſome degree of reſiſtance felt to the raiſing up of the uterus; ſo that it can only be by thoſe who are perfectly accuſtomed to diſtinguiſh the feel of the unimpregnated uterus that this can be determined. As the pregnancy advances the caſe will become clearer, and we ſhall be able, by degrees, to determine deciſively that the conception is not uterine; and, by the external feel and appearance, to form an opinion of the actual ſituation of the fœtus.

We are told that theſe conceptions ſometimes take place under the external membrane of the ovarium or tube, and that in theſe caſes the dilatation may go on with much leſs difficulty to the mother; but we want accurate diſſections, and are frequently at a loſs to underſtand thoſe writers who attempt to be the moſt particular in their accounts of ſuch caſes. Fortunately, however, theſe diſtinctions ſignify very little in practice, as all theſe extraordinary variations of nature, in this buſineſs, are almoſt equally deplorable and unmanageable by art, till nature kindly points out how ſhe chooſes to be relieved. And it has been uniformly obſerved in all theſe caſes, that at the natural term of pregnancy there is always an effort to expel the child, which, in ſome caſes, is frequently and violently repeated. And it has been alſo obſerved, that at whatever of the later months the child dies, there commonly follows a flow of milk to the breaſts.

From the caſes I have adduced, I think it appears clearly that a child, remaining in the cavity of the abdomen, is ſo far from being neceſſarily a fatal accident, that it does not even prevent future pregnancies, and conſequent natural births; nay, we farther know, from a caſe communicated by Dr. Steigerthall, in the Philoſophical Tranſactions *, that a woman has lived in good health to the age of ninety-four with a full grown fœtus in her abdomen for the laſt forty-ſix years of her life, during which period ſhe bore two other children. But what is ſtill more extraordinary, we have good reaſon to be aſſured that women have not only for a conſiderable time ſurvived, but even ſometimes recovered, by the powers of nature, after the child has eſcaped through a rupture of the uterus. I am, therefore, much inclined to believe, that when this

* Vol. XXXI. p. 126.

accident accident happens at any period of pregnancy previous to the complete dilatation, or rather to the eafy dilatability of the natural paffages, the mother will have a better chance for life, if left to the refources of nature, affifted by palliative remedies, than by a fpeedy and violent dilatation of the parts, and an extraction of the child through a lacerated uterus, which is likely ftill to fuffer more by fuch an operation in a conftitution much weakened, and at that time highly irritable from pain, anxiety, and terror.

I have myfelf been called in to ten cafes of ruptured uterus, and have before me the account of many others attended by gentlemen of the firft abilities and experience. Of thefe much the greater number were delivered very foon after the rupture took place *; but in no inftance have I had reafon to believe that the mother furvived a longer time than fhe would have done if left entirely to nature; and were I to prefume to conjecture from the cafes of this kind that have occurred to me, I would fay hardly fo long.

* Delivery was effected in all the cafes where a fpeedy death did not render it unneceffary.

I could eafily give a number of cafes to which I have been witnefs, in fupport of this opinion, but none ftronger than the one which happened to a patient of my late worthy friend Dr. Bromfield, whofe caution, fagacity, and experience, were well known to many of his brethren. Whilft he was patiently attending the natural labour (which had not lafted long) of a lady who had a well-formed pelvis, and had been the mother of feveral living children, on a fudden his patient gave an uncommon fcream, and, on examination, he found that the uterus had given way, and the child efcaped beyond his reach. This happened when the paffages were fully dilated, and when the head of the child was at the os externum, fo that the delivery feemed to be nearly accomplifhed. No very alarming fymptoms immediately followed the accident; and the patient was very cautioufly, and with great eafe, delivered of a child that feemed to have been fome time dead. This was done by the advice of five phyficians of experience, three or four hours after the accident, but fhe furvived the delivery only thirty-fix hours.

It may, however, be confidered as a good general doctrine, that in all cafes of labour rendered rendered dangerous by flooding, convulſions, or rupture of the uterus, the delivery of the child, when the ſtate of the natural paſſages will allow its being done without violence, is a deſirable circumſtance, on account of the prejudice of the world, and of the patient herſelf, in favour of delivery; beſides the better chance of recovery it may ſometimes give. But from all I have been able to obſerve, during a number of years, I am fully inclined to believe that a violent and ſpeedy delivery is not the ſafeſt practice; and though in the caſes of hæmorrhage there may be more frequent and urgent reaſons for it, yet I am certain that great diſcrimination and caution are neceſſary even in theſe; but in caſes of convulſions I can ſpeak with more certainty.

In October, 1779, I had the honour to lay before the Society of Phyſicians, Licentiates of the College of London, an eſtimate of the events of convulſion caſes, taken with tolerable accuracy from moſt of thoſe on record, as well as from a pretty large number I had myſelf been witneſs to. From theſe it appeared that a greater number of thoſe women recovered who had not been delivered, by violent dilatation, than of thoſe who had; and that delivery, livery, sometimes, had not even cured the convulsions, much less saved the patient.

With regard to ruptures of the uterus, I was first induced to be of this opinion by a remarkable instance of such an acccident which occurred spontaneously, at an early period of labour, before the os uteri was dilated, and at a time when nature seemed to have exerted so little violence as to render it very difficult even to conjecture how it could have happened.

This very unfortunate and unexpected occurrence induced me to estimate the consequences of all the variety of the accidents of this kind, which either my reading, my information, or my practice, could furnish me with. From this inquiry I was led to believe that my patient (the first I had then heard of, in this country, who had been known to survive such an accident for so long a time as twenty-six days) would have had a better chance of recovery, if, according to the opinion of the late Dr. Hunter, Dr. Bromfield, and Mr. Graves, joined to my own, she had been left to the resources of nature; instead of having a child, known to be dead, dragged, through the lacerated uterus, by the exertions necessary to effect the dilatation and extraction: but, unfortunately, we were overover-ruled. Her ſymptoms, not apparently mortal before, were certainly not relieved by delivery. She died on the twenty-third day after the extraction, and her body exhibited all the appearances of peritonæal inflammation, which there is the greateſt reaſon to ſuſpect took place after that period.

As there are repeated inſtances to prove that a rupture of the uterus, near the full time, is not always, of itſelf, neceſſarily a fatal accident, I hope I am juſtified in preſuming to ſuppoſe, that had my patient been kept perfectly quiet, the non-naturals been attended to, and occaſional ſymptoms obviated, ſhe would have had a better chance from the reſources of nature.

It has not yet appeared from any one caſe I am acquainted with, that the mother's life has been ſaved, by a delivery, where a violent dilatation of the natural paſſages was neceſſary; but, on the contrary, that almoſt all have died who have been delivered ſpeedily, even when this has been done with very little trouble. Whereas, on the other hand, ſeveral have ſurvived when they have been entirely let alone. But the fact really is, that thoſe caſes in which an opportunity for deliberation is allowed of, are unfortunately as rare as they are dangerous, the accident moſt commonly taking place at a time when the labour is very far advanced, and when the dilatation of the os uteri is totally or nearly completed; in which ſituation, if we find it can be done with eaſe, there are many reaſons, prudential as well as ſcientific, to determine us to relieve the patient's mind, and the minds of her friends, as well as to free the abdomen from this extraneous body. But were we even convinced that her chance for life would be better by refraining to add violence to violence, this oppoſition to general prejudice can only be effected in patients whoſe apprehenſions we can quiet, with friends whoſe reaſons we can convince, and by the authority of reputations ſo eſtabliſhed for judgement, experience, and humanity, that no blame can be incurred, whatever may be the event.

To thoſe who have conſidered this ſubject it will appear clear, that no part of this reaſoning will apply to any of thoſe caſes where a laceration of the vagina, or of any part of the cervix, takes place after the complete dilatation of the os uteri, ſo that no violence becomes neceſſary in order to introduce the hand: and this appears to me to have been preciſely the caſe in almoſt all thoſe fortunate inſtances where the patients recovered after a ſpeedy delivery. It is perfectly well eſtabliſhed, by experience, that lacerations of the vagina and cervix uteri, though always dangerous, do frequently happen with impunity; and this has been confirmed to me by inſtances to which the late Doctors Hunter, Harvie, John Fordyce, and ſeveral other eminent practitioners, have been witneſs; and I could eaſily quote inſtances of recovery after lacerations of the uterus, very ſimilar to thoſe mentioned by Peu* and Dr. Hamilton †; or to that where the cervix uteri had been violently torn, as deſcribed by an eminent practitioner in Dorſetſhire in a letter to the late Dr. Hunter, which the latter communicated to the Society of Phyſicians, and which having never been publiſhed, I ſhall here mention more particularly, and as a farther proof of what I have aſſerted.

This caſe happened in the year 1777. The labour had been tedious and fatiguing, and the os uteri being very ſlow in dilating, the forceps were prematurely attempted to be applied, and the firſt blade was violently introduced through

* Pratique des Accouchemens, p. 341.

† Outlines of Midwifery, p. 344.

the undilated mouth of the uterus. In the raſh and forcible attempt to introduce the ſecond blade, it was forced through the ſubſtance of the cervix uteri, into the cavity of the uterus, at that part where it is inſerted into the vagina, and the blades being locked, the head was attempted to be extracted by main force. The torment occaſioned by this violent and raſh operation was too excruciating to be endured. Another practitioner was immediately called in, who, diſcovering the head of the fœtus protruding through the artificial opening, and enlarging the laceration towards the fundus uteri at each return of pain, thought proper to divide the os uteri, and to lay both openings into one. By a few more ſtrong pains the patient was delivered, and ſhe recovered without a ſingle dangerous ſymptom of any kind ſupervening.

That lacerations of the vagina and cervix uteri (which, at the period of complete dilatation, are to be conſidered as one canal) ſhould be much leſs dangerous than ſuch as happen higher up, may be probably accounted for by the much freer diſcharge which the blood, ſerum, and pus, can have out of the body.

I need not add to the number of caſes, in which it has been found that the cervix uteri may be be injured with impunity, thofe very remarkable ones in which there has been a neceffity, from fome mal-conformation, to open the os uteri, by incifion, at the time of labour, in feveral of which we know children have been happily delivered with perfect fafety to the mother.

It is by no means neceffary to enter into any reafoning refpecting the fituation of the rupture in the cafe fo accurately defcribed by Dr. Douglas*, or to determine whether Mr. Goldfon is right or wrong in fuppofing † this cafe to have been only a rupture of the vagina. One circumftance, however, remarkably diftinguifhes the cafe which occurred to Mr. Goldfon from that defcribed by Dr. Douglas. In the former, the bladder was ruptured, and in the latter it was not, which, I think, renders it more than

* Obfervations on an extraordinary Cafe of ruptured Uterus. 8vo. London, 1785. — See alfo Vol. VI. of this Journal, p. 91.

† In a work, entitled "An extraordinary Cafe of lacerated "Vagina, at the full Period of Geftation; with Obfervations, "tending to fhew that many Cafes related as Ruptures of the "Uterus have been Lacerations of the Vagina." 8vo. London, 1787.

probable, probable, that though both the ruptures might be tranſverſe and low down, yet that in the caſe deſcribed by Dr. Douglas, the rupture was higher than in the other. From the account Dr. Douglas has given of this caſe, I find it to have been of that kind that required n[illegible] artificial dilatation to effect the delivery, his hand having been in the cavity of the abdomen, and in contact with the inteſtines and bod[illegible] of the child, before he knew preciſel[illegible] what the caſe was. In that ſituation there could be neither occaſion nor time for heſitation, and he certainly decided right in bringing the feet down with him. Nothing could be better managed, or more fortunate; but it requires no reaſoning to prove that neither from this caſe, nor from any of the other fortunate ones before mentioned, can a rule of practice be derived as to the mode of treatment when the rupture takes place whilſt the parts remain in a great degree undilated; for which reaſon what I have ſuggeſted, as the ſafeſt method in ſuch ſituations, remains preciſely as if no ſuch caſes had ever happened.

The practice of ſpeedy delivery is neither new nor untried. It is the obvious, and it has been the common practice; but it has generally rally been unfuccefsful *: and the contrary method, as it oppofes common prejudices, which are always on the fide of delivery, has been hitherto (if I may be allowed the expreffion) very unpopular. I would only wifh to awaken the minds of the judicious and candid to a confideration of the moft probable chance of life to the mother in fo defperate a fituation. I hope I need not recommend the refraining from all operative methods, when the rupture takes place at any period of pregnancy, whilft the uterus remains fealed up, as it were, and whilft the cervix uteri remains unobliterated; though it is difficult to fuppofe that any one, poffeffed of even the flighteft knowledge of the practice of midwifery, would venture to attempt to force open the uterus at fuch a period, yet fuch attempts are much to be feared from the daring

* In the Collectanea Societatis Havnienfis, Vol. II. p. 203, there is a remarkable cafe of a fpontaneous rupture of the cervix uteri and vagina on the right fide, through which the child efcaped, and in which, delivery, though immediately effected, did not long protract the mother's life, who died the next day: and we find a fecond in the fame volume, p. 326, where the rupture feems to have been occafioned by violent attempts to turn the child, and where delivery, though immediately effected, did not obviate the moft dreadful fymptoms, which came on fpeedily, and, notwithftanding the moft judicious exertions, became fatal in three months.

and inexperienced, if ſpeedy delivery be inculcated as giving the beſt chance of recovery in all caſes where the uterus is ruptured. There can be no doubt that by an over caution, approaching to timidity, patients may ſometimes be ſuffered to die who would probably recover by a bolder practice. But this is not the common fault of the young and inexperienced; for in them we more generally find a natural propenſity to activity, and a deſire to aſſiſt, which, in midwifery, is infinitely the more dangerous extreme; and it is perfectly well known that caution and the leaving much to nature, which are ſo eſſentially neceſſary in this practice, are the uſual effects of age and experience, and are certainly the beſt general rules, though not without exceptions with reſpect to the latter, which ought only to be made from the dictates of ſound judgement, matured by accurate obſervation.

I will venture to go ſtill farther, and to declare, though with the utmoſt diffidence, that if I could be aſſured of the child's being alive, I would join in opinion with Bartholine, Aſtruc, Roederer, Plenck, Levret, Baudelocque, and many others, that it appears to be a ſafer method, and likely to give the woman a better chance of recovery, to divide the parietes of the abdomen, and extract the child in that way, than to riſk a forcible dilatation and increaſed laceration of the uterus. I will beg leave to ſubmit my authorities for hazarding this opinion with reſpect to a practice which has not, ſo far as I know, been adopted in this country. Firſt, then, Bartholine ſays, "Facilior omni-

"no hic naſcendi modus periti chirurgi manu

"adminiſtratus, qui noſtras fæminas terret, ſec-

"tionis iſtius ignaras, minorique dolori junc-

"tus, quùm citius levi vulneri abdomen aperi-

"atur, quam naturales partes dilacerentur *."

Aſtruc † tells us that a German phyſician (whom he does not name) has written a very good diſſertation on ruptured uteri, in which he propoſes to extract the child by dividing the integuments of the abdomen; and this method Aſtruc highly approves of in caſes where the child has previouſly eſcaped into the general cavity. He aſſures us that if this operation is performed as ſoon as the mother can bear it, and when ſhe has recovered the immediate flurry of the accident, it will certainly ſave the child, and very probably the mother alſo; it being well known that ruptures of the

* De inſolitis partus human. viis, p. 107.

† L'Art d'Accoucher, p. 291.

uterus are not of themſelves always incurable, and this operation being no more than a ſimple inciſion of the integuments.

Roederer, in his Elementa Artis Obſtetriciæ *, expreſſes this opinion very ſtrongly. "Quoties integer fœtus, vel ſaltem cùm capite truncus, prouti frequentiſſime accidit, "extra uteri cavum propulſus eſt, ſola abdominis *matura apertio* matri forſan et fœtui "vitam ſervare poteſt. Utero, contuſione, "gangræna ſphacelove, ut ſolet, corrupto, "debitis remediis, utcunque poterit, medicus "proſpiciat."

Plenck is of the ſame opinion : — "Si vero "fœtus per uteri vulnus invenitur toties in "abdomine elapſus, tunc *gaſtrotomia* indicaretur, ut fœtus poſſet educi; at ut plurimum peſſimorum ſymptomatum, et mortis "inſtantis præſentia quemlibet ab operatione "deterrent †."

Van Doeveren ‡ adviſes, if the child eſcapes through the ruptured uterus alive, that it ſhould immediately be ſaved by opening the parietes of the abdomen.

* Cap. xxv. §. 767.

† Elem. Art. Obſ. p. 129.

‡ Obſ. Acad.

It

It would be eaſy to multiply authorities, if theſe could prove any thing. Suffice it to ſay, that this is the preſent method of practice in France, Germany, and the Low Countries, eſtabliſhed on what is there conceived to be the teſt of experience; in proof of which we need only look into M. Baudelocque, one of the lateſt and beſt of the French writers, and at this time one of the moſt popular teachers and practitioners at Paris. In his "Art des Ac-"couchemens," treating of the caſe where the child has only made its way partially through the uterus, he ſays, that dividing the integuments of the lower belly is as manifeſtly indicated in caſes of this kind, as in thoſe where the child has puſhed its way entirely into the general cavity. We can, he obſerves, conceive but too clearly to what danger we ſhall expoſe a woman in endeavouring to turn a child, of which the greateſt part has already paſſed into the cavity of the abdomen, and the reſt remains ſtrongly graſped by the lacerated uterus. Some modern ſurgeons, he adds, leſs timid than Saviard and many others, have already, by gaſtrotomy, preſerved the life of the mother, and, had they been called in time, would, he thinks, moſt probably have

ſecured that of the child alſo. In proof of this aſſertion, he quotes a caſe communicated in the *Journal de Medecine* for May, 1768, by M. Thibault de Bois, Surgeon at Mans, of a woman through whoſe uterus the infant had burſt when the labour was far advanced, and every thing promiſed a happy delivery. On the very ſame day M. Thibault, in the preſence of four of his brethren, extracted the child, which was dead, along with the placenta, through an inciſion of the abdominal integuments. The woman recovered without any bad accident, was able to return thanks at church on the thirtieth day after the operation, and had continued in perfect health, though at the time of his writing the account ſhe had not menſtruated. M. Baudelocque farther adds, that he makes no doubt but the Royal Academy of Surgery will publiſh a ſimilar caſe, communicated by a ſurgeon of Orleans, on whom they beſtowed one of their leſſer medals ſome years ago, he having been happy enough (and with perfect ſafety to the mother) to ſave, by this operation, a child, which, after a long labour, had burſt into the cavity of the abdomen. The ſame writer farther ſays, that this operation is not only neceſſary to give a paſſage to the child, child, but alſo to the blood and water extravaſated in the lower belly. He conſiders it as a much eaſier operation than the Cæſarean, and the caſe as not more dangerous, as wounds in the uterus, though made by rupture, are not eſſentially mortal, and do not require more care than when made by a cutting inſtrument. Speaking of La Motte and others, who have extracted the child through a ruptured uterus, by the natural paſſages, he ſays, "Although "this operation is not always impoſſible, yet "he does not quote theſe caſes as examples "that ought to be followed."

M. Jacob, in his Ecole Pratique des Accouchemens*, publiſhed in 1785 at Ghent, recommends this operation as the only thing to be done to ſave both mother and child, when the latter has eſcaped into the cavity of the abdomen, and inſinuates that in that part of Europe it has not been found dangerous †.

M. Tenon, Member of the Royal Academies of Sciences and Surgery, and Surgeon to

* Page 215.

† May not the caſe mentioned in the London Medical Journal, (Vols. VI. and VII.) of the negro woman who performed the Cæſarean operation on herſelf, and an inſtance at Hamburgh, of its being performed by a bull's horn, ſhew us that in ſome conſtitutions this operation is not fatal?

the the Salpetriere Hofpital at Paris, equally eminent for his learning and long experience, informed me, when lately in this country, that, in all extra-uterine cafes, and in the greater part of thofe where the child has burft, either wholly or partially, through the uterus, the French accoucheurs conftantly extract it through the divided parietes of the abdomen, and that they confider this as by much the fafeft practice. He is of opinion that the Cæfarean operation is lefs fuccefsful in this country than it is in France, becaufe we defer it too long; and he affured me that fince their firft practifing this operation, in the time of Bauhin, feventy-eight women have been faved by it at the Hotel Dieu of Paris. From this account, which I have repeatedly received from M. Tenon, added to thofe inftances of fuccefs which I have had occafion to mention in my inquiry into the events of extra-uterine cafes and ruptures of the uterus, I read with lefs furprife feveral recent accounts of extraordinary recoveries after operations feemingly the moft defperate, which have been publifhed in the French Memoirs and Journals. For inftance, in the *Journal de Medecine* for Auguft, 1786*,

* Page 201.

we have an account of the total extirpation of the uterus; in the Memoirs of the Royal Medical Society at Paris *, another of the total extirpation of the ovarium; and in the same work we have an account of two successful Cæsarean operations †. In spite of all this, however, when I consider how very unsuccessful the Cæsarean operation has been in this island, under the best management, and when I farther consider how extremely hazardous we find all exposures, of the abdominal cavity, to the external air, to be, I own I think that even the simpler excision I have been speaking of, ought never to be had recourse to but where there is reason to hope, from the absence of inflammation, and other dangerous symptoms, that the mother's life may be saved by it; or where there is almost a certainty of preserving the child: as this expedient appears to me, while the natural passages are undilated, to be not only a more probable method to save the child, but even a less hazardous one for the life of the mother. And though it must be acknowledged that, in this country at least, the preservation of the mother seems most likely to be effected by abstaining

* Vol. IV. page 296.

† Vol. II. pages 236 & 241.

from all violence, yet it may deserve consideration whether the chance this may give of a speedy cure, may not be preferable to that long-protracted misery, and distress, which the unhappy woman must undergo from a child remaining long in the cavity of the abdomen, even if she should be fortunate enough to survive its expulsion through the parietes of the abdomen, or by the intestines, so many instances of which I have had occasion to relate.

Though sudden ruptures of the cervix uteri may be often less dangerous than those near the fundus, yet there is one cause of tranverse division of the uterus, which, whether delivery be performed or not, is, I believe, always fatal—I mean where the texture of the uterus is destroyed, and inflammation and mortification brought on by the pressure, during labour, of the projecting process of the os sacrum, or sharp ridges of the os pubis, or ilia, in a narrow pelvis, against the head or breech of the child. Of this I saw a very remarkable instance in August, 1786. A woman, with a narrow pelvis, had, at the full time, a breech presentation, and though she did not suffer the strong compressing pains of labour more than twelve hours, yet, at the end of that period, and before the os uteri was completely dilated, the whole whole fore part of the cervix uteri ſeparated from ſide to ſide. This was owing to the preſſure of the large breech of the child againſt the ſharp ridges of the ilia and pubis. The child paſſed into the general cavity of the abdomen, and a foot preſented.

In leſs than two hours after the accident the child was extracted, dead, and with no other difficulty than what was occaſioned by the narrowneſs of the pelvis; but the woman ſurvived the delivery only five hours.

The poſterior part of the cervix uteri was found to be worn through by a large projection of the ſacrum, which was angular and ſharp, but not ſo much ſo as the internal ſuperior ridge of the os pubis and ilia, which reſembled the edge of an ivory folder, and had cut the uterus through in the manner a ligature does a polypus.

In all caſes where diviſion of the uterus is occaſioned by preceding compreſſion and mortification, I conſider the fate of the woman as determined before that accident takes place. This may explain how comparatively ſhort a time ſome women can bear the compreſſion of a head or breech in the narrow pelvis to what others can, and how ſudden the fate of many muſt be after ſuch a rupture. This ought to lead us, if poſſible, to aſcertain, as well as we can, in the earlier period of labour, not only the ſize, but the conformation of the narrow pelvis, and whether there be any ſharp angles, in which caſe compreſſion is always to be dreaded.

To mention that we are ever to watch attentively, and to obviate, by every poſſible means, all cauſes of inflammation during labour, or to ſay that inflammation begun before or during labour, is always highly dangerous, and commonly fatal, is ſaying only what all experienced practitioners muſt have obſerved. But to awaken the attention of the unexperienced to the ſtate of the pulſe and concomitant ſymptoms, in laborious caſes, is important, as we muſt often be determined by theſe as much as by the ſituation of the child, what is proper to be done. If ever a woman has any chance from nature, in a ruptured uterus, it muſt be where no inflammation has preceded the accident ; and the prevention of ſuch irritation as is capable of exciting this, is the preciſe reaſon why I think her being let alone, or the dividing the integuments of the abdomen, is giving her the beſt chance.

These obfervations being only offered with a view to the improvement of the practice of midwifery, I have ftill one farther caution to add, and that is with regard to a method propofed by feveral refpectable authors for preventing ruptures of the uterus. They tell us, that when from the fenfation of exquifite pain in any one particular part of the uterus, or when there is any particular obftruction of the paffages which may probably give occafion to long-protracted, violent, and ineffectual throes, or when there is any thing that appears to us fo peculiar in the nature of the labour, or the conftitution of the woman, (which it is allowed to be very difficult to defcribe) that fhall induce us to fufpect a rupture of the uterus likely to happen, we are directed in that cafe to precipitate the delivery by turning the child.

Now, befides the general objections, which always fubfift againft turning a child, where the head prefents in a contracted uterus, after the waters have been fome time paffed off; as I prefume the fymptoms threatening rupture can never be obferved, or even fufpected, before the child is clofely embraced by the ftrong contractions of the uterus, I fhould fear the attempt to dilate it, and turn the child in fuch circumſtances, would be preciſely incurring the greateſt danger of the very accident we mean to prevent.

Every body knows that to turn a child, in a ſtrongly contracted uterus, is at all times attended with ſome danger of a rupture; how much more muſt it be ſo, when, from any pre-indicating ſymptoms, or circumſtances, however ſtrong, we can be led to ſuppoſe that a rupture is in danger of taking place. I myſelf know an inſtance where a ſurgeon, in attempting to graſp the feet of the child when the arm preſented, happening only to cloſe his hand in the uterus, found the latter immediately give way. Though the woman was at the ſame inſtant delivered, and no very violent ſymptom immediately ſupervened, yet ſhe died in a few days.

I am, therefore, clearly of opinion, that even if the preſaging ſymptoms were more deciſive and determinate than I think they ever can be, this method of prevention is too hazardous to be attempted; but, equivocal as they muſt be, I think it is ſtill leſs to be juſtified, let the internal evidence be ever ſo ſtrong.

I am ſtill the more confirmed in this opinion by a recent caſe which happened in my own practice, practice. A woman, with a well-formed pelvis, mother of two living children, when in a natural labour, at the full time, with a very large head presenting, screamed out, and complained loudly of an excruciating pain in one particular part of the left hypogastric region; at the same time that she seemed to be all over affected with spasms, so as to give me very serious apprehensions that she might either fall into convulsions, or suffer a rupture of the uterus in that particular part. I, therefore, by cautious dilatation, and by assisting the effect of every pain with the vectis, in as short a time as I could, with safety to the parts, delivered her of a large living child, and she recovered without the least accident. I think in any of these cases, with the head presenting, whilst the child is alive, we can be justified in promoting the labour only by a method similar to what I have mentioned, or by the forceps, if the head be within reach. If these symptoms occur before the dilatation of the os uteri, bleeding, in plethoric habits, or warm bathing, and opium, in case of irregular spasm, may be useful. I can hardly suppose a case, with the head presenting, in which it would be expedient to turn, after the waters have been passed off off long enough to allow the uterus to embrace closely the body of the child, unless in that of a very dangerous flooding, in cases where the funis presents, and its pulsation is perceptible, or where the uterine contractions are too inert to act properly in the expulsion of the child: in neither of which cases is the contraction of the uterus usually so strong as to render the necessary dilatation very dangerous.

I have undesignedly extended this paper to a length I did not think of when I began it; I must, therefore, delay some farther observations I have to make on ruptures of the uterus till a future opportunity.

*St. Martin's Lane,*
Sept. 20th, 1787.

